# Recent advances in rational approaches for enzyme engineering

**DOI:** 10.5936/csbj.201209010

**Published:** 2012-10-22

**Authors:** Kerstin Steiner, Helmut Schwab

**Affiliations:** a ACIB GmbH, (Austrian Centre of Industrial Biotechnology), c/o TU Graz, 8010 Graz, Austria; b Institute of Molecular Biotechnology, TU Graz, 8010 Graz, Austria

**Keywords:** protein engineering, rational design, *de novo* enzyme design, structure-guided engineering, promiscuous enzymes, artificial metalloenzymes

## Abstract

Enzymes are an attractive alternative in the asymmetric syntheses of chiral building blocks. To meet the requirements of industrial biotechnology and to introduce new functionalities, the enzymes need to be optimized by protein engineering. This article specifically reviews rational approaches for enzyme engineering and *de novo* enzyme design involving structure-based approaches developed in recent years for improvement of the enzymes’ performance, broadened substrate range, and creation of novel functionalities to obtain products with high added value for industrial applications.

## Introduction

The synthesis of chiral compounds is a challenge in chemistry. Enzymes are often superior to non-enzymatic catalysts concerning effectiveness, enantioselectivity and environmental friendliness. However, although natural enzymes are versatile biocatalysts catalyzing a wide range of chemical reactions, they are evolved towards the needs of their natural role. Thus, they are not available for many of the important conversions and substrates relevant for industry and do not fulfill the manifold requirements on enzymes used in industrial biotechnology. Enzymes should have high activity as well as high specificity and enantioselectivity towards frequently very challenging substrates. Moreover, they need to be stable during storage and resist a variety of - sometimes harsh - reaction conditions such as elevated temperature, extreme pH, high substrate / product concentrations and organic solvents. Thus, to fulfill all these criteria enzymes are nowadays routinely optimized by enzyme engineering for application in organic synthesis [1]. The published examples are uncountable and the interested readers are referred to numerous excellent reviews and book chapters available on protein engineering and the application of engineered enzymes in organic synthesis [2–9].

This article specifically reviews rational approaches for enzyme engineering and *de novo* enzyme design involving structure-based methods developed in recent years for improvement of the enzymes’ performance, broadened substrate range, and creation of novel functionalities to obtain products with high added value for industrial applications.

## Current status of protein engineering

**Figure 1:**
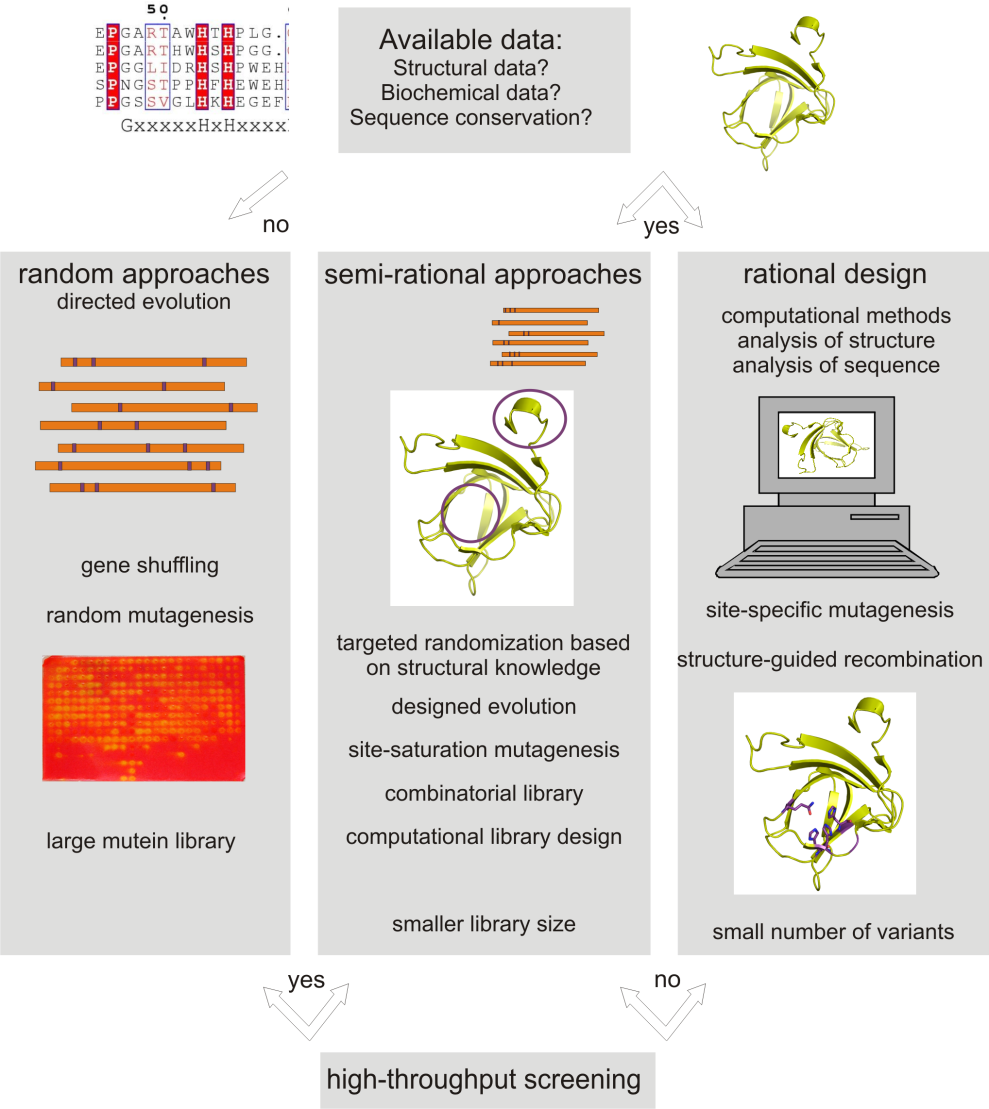
Overview of approaches for protein engineering by random, rational and combined methods.

Generally there are two main strategies for protein engineering: directed evolution and rational design, which can be combined to semi-rational design or focused directed (designed) evolution (Figure 1).

Directed evolution can be achieved by two major approaches, either by randomly recombining a set of related sequences (e.g. gene shuffling), or by introducing random changes in single protein sequences (e.g. error-prone PCR). The advantage of directed evolution is that no structural information is needed and that variations at unexpected positions distant from the active site can be introduced. However, usually the changes are small and several rounds of evolution have to be applied and thus a high number of variants have to be screened, which is time and labor consuming and requires cheap, fast and reliable high-throughput assays.

With the availability of an increasing number of protein structures or reliable models, biochemical data and computational methods, enzyme engineering is developing more and more from random approaches (directed evolution) to semi-rational or rational (data-driven) design. In rational design biochemical data, protein structures and molecular modeling data are evaluated to propose mutations, which are introduced by site-specific mutagenesis. One of the advantages of a rational design approach is an increased probability of beneficial mutations and a significant reduction of the library size and thus less effort and time has to be applied for the screening of the library. This is especially advantageous if no high-throughput assay system is available.

Semi-rational design combines advantages of rational and random protein design creating smaller smarter libraries based on knowledge derived from biochemical and/or structural data [10]. An example for a semi-rational approach is CASTing (combinatorial active site saturation test), which uses the information derived from e.g. structural data to identify amino acids in interesting regions (e.g. active site), which are then mutated randomly or by site-saturation mutagenesis one by one or in combination [11–13, 8]. Random combination of mutations or correlated mutations at targeted positions can result in synergistic effects that might have been missed in single site-specific mutagenesis. However, these combinatorial approaches increase the library sizes tremendously and various computational methods have been developed in recent years, that help to decrease the library size by screening of virtual libraries and eliminating mutations predicted to be unfavorable for the protein fold [14–18].

## Learning from natural diversity and conservation

### Structure-guided consensus approach

In structure-guided consensus approaches sequence-based and structural data are combined. Generally, the consensus approach is based on the hypothesis that consensus amino acids of a sequence alignment contribute more than average to the fitness of the protein than the non-consensus amino acids. They proved to be favorable for the protein during natural evolution during which unfit variants are eliminated. Thus, changing non-consensus amino acids to consensus amino acids should improve e.g. thermostability (for details see [19]). A proof of concept was given after several rounds of this method, when the unfolding temperature of a fungal phytase was increased by more than 30°C to astonishing 90.4°C [20]. This approach was also successfully used to increase the thermostability of a cellulosomal endoglucanase, Cel8A, from *Clostridium thermocellum* 14-fold at 85°C without loss of catalytic activity [21]. However, after detailed analysis of the mutants, it turned out that of the eight identified consensus positions one single mutation (G283P) was sufficient to produce a thermostable variant. Glycine to proline mutations have been described before to increase the thermostability, however only at sites carefully selected [22, 23]. Thus, as not all of these consensus mutations actually contribute to stability and some might interfere with the enzymes’ activity, additional factors should be implemented. Structural features, such as the distance to the active site (improvements in stability in most cases require mutations further away from the active site, whereas changes of selectivity and activity usually target directly the active site), avoiding destabilization of helices or breaking existing hydrogen-bonds or salt-bridges, have to be considered [24–26].

By applying this structure-guided consensus approach, Bommarius and his group were able to increase the thermal stability of a penicillin G acylase (PGA) [25] and a glucose dehydrogenase (GDH) from *Bacillus subtilis* [27] by preserving the enzymes’ activities. In both cases about 50% of the variants showed increased thermostability. The three most stable mutations of GDH were successfully applied to two other GDHs from *B. thuringiensis* and *B. lichenformis*. Moreover, they showed that the most thermostable variants of GDH from *B. subtilis* were also more stable in high-salt solutions and homogeneous aqueous-organic media [28]. The same group combined the consensus approach with the B-fit (B-Factor Iterative Test) method to improve the thermostability of an α-amino ester hydrolase from *Xanthomonas campestris* resulting in a quadruple mutant (E143H/A275P/N186D/V622I) with 7°C improvement and 1.3-fold activity compared to wild-type [29]. In the B-fit method positions that display the highest flexibility (highest B-factor) in an enzyme's crystal structure are subjected to site-saturation mutagenesis [30–33]. The library size can be reduced by limiting the variation at the chosen positions to amino acids that are frequently present in an alignment using a consensus approach. An extension of the consensus approach is the combination of a sequence alignment with phylogenetic trees to identify the consensus sequence of a common ancestor [34]. It is thought that this common ancestor should be more stable due to the harsh environmental conditions during ancient times [35] and in addition this sequence might show promiscuous enzyme activity as it displays the turning point towards two separate enzymatic activities [36] (see below). Ancestral residues can also be introduced into modern sequences thereby combining the advantageous properties of the ancient (e.g. thermostability) and the modern proteins (e.g. activity, substrate range, selectivity) [37, 38].

### 3DM database

**Figure 2:**
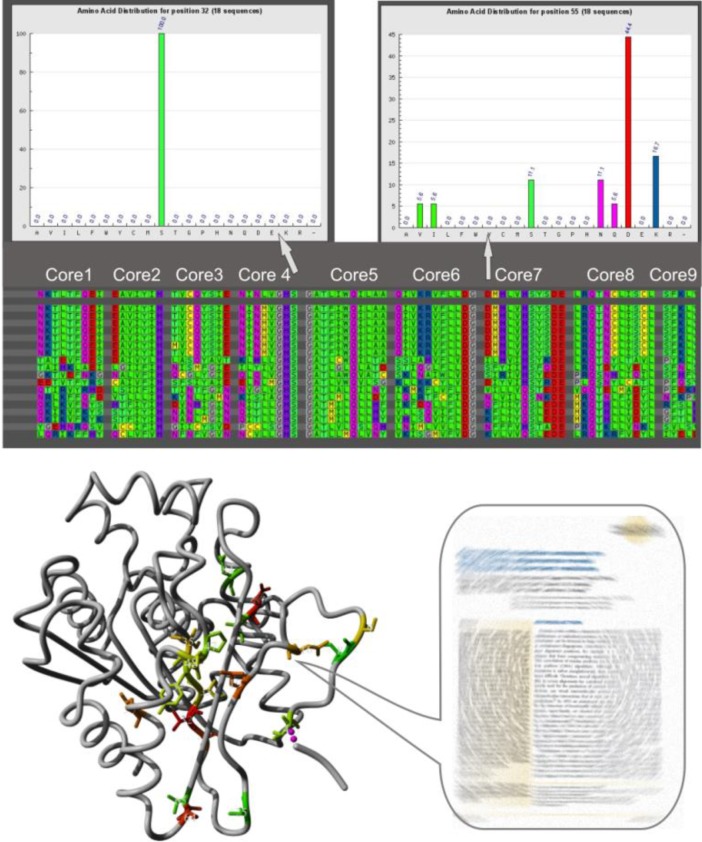
**Exemplified features of 3DM databases.** 3DM databases link structural data, sequences and biochemical data (activity, stability, protein-ligand interaction, reported mutations). Middle: structure-sequence alignment of a subfamily, Top: amino acid distribution at chosen positions of the alignment, Bottom: mutations described in literature are linked to and depicted in a structure.

3DM databases are protein super-family platforms that combine different types of protein-related data including structures, multiple sequence alignments, conserved amino acids, (correlated) mutations, protein-ligand and protein-protein contact information. They are linked to literature containing mutations and biochemical data (Figure 2) [39–41]. 3DM databases can be applied for different purposes. Based on analysis of a 3DM database containing proteins of the α/β-hydrolase fold superfamily, the enantioselectivity (from E = 3.2 to E = 80) and activity (up to 240-fold) of an esterase from *Pseudomonas fluorescens* was improved by mutating four residues near the active site [42]. In addition, the thermostability of the same esterase was also improved in combination with the B-fit method [43]. Using the same database, a variant esterase from *Paenibacillus barcinonensis* with increased activity and enantioselectivity for the synthesis of tertiary alcohols was found [44]. Combination of the B-fit method with a 3DM database containing sequences of the glucosidase family 13 helped to identify positions where mutations led to increased thermal stability of a sucrose phosphorylase from *Bifidobacterium adolescentis* [45]. This study also revealed a synergistic effect of the two most flexible positions, as only pairwise mutations showed the desired outcome. In the most successful variant they introduced additional salt bridges. Analysis of a 3DM database for FAD-linked oxidases revealed, that almost all oxidases in the vanillyl-alcohol oxidase (VAO) fold subfamily contain a Gly or a Pro at a certain position, whereas other members that do not react with oxygen, have a different residue. Changing the corresponding residue in an L-galactono-γ-lactone dehydrogenase (GALDH) from Ala to Gly resulted in increased oxygen reactivity thus converting the dehydrogenase into an oxidase [46]. Very recently, a 3DM database for methyltransferases helped to identify amino acid residues relevant for catalytic function and binding of the cofactor S-adenosylmethionine, which were confirmed by mutagenesis analysis [47].

### Structure-guided recombination

Another approach to improve the thermostability of proteins uses SCHEMA structure-guided recombination. SCHEMA is a computational algorithm, which estimates the disruption caused when amino acid residues that interact in the structure of a protein are recombined in chimeras. Sequences with low sequence similarity can be shuffled and the chimeras are rated according to the disruption caused [48–50]. With this method the thermostabilities of cellobiohydrolases class I, cellulases and cytochrome P450s were improved [51–53]. In addition, chimeric P450s were able to accept substrates that were not accepted by the parent protein, thus broadening the substrate range [54]. Recently, the thermostability of a Baeyer-Villiger monooxygenase (BVMO) phenylacetone monooxygenase was combined with the broader substrate range of other BVMOs by structure-guided subdomain exchange [55]. Interested readers can find more information about ‘Protein design with fragment databases’ in the recent review by Verschueren and coauthors [56].

## Active site (re)design

### Promiscuous catalytic enzyme activities

Generally, enzymes can be promiscuous concerning their reaction conditions (reaction condition promiscuity), their substrate range (substrate promiscuity), show an additional activity in the same active site (catalytic promiscuity) or due to a second active site (alternate site promiscuity) [57]. Some enzymes display a minor catalytic activity (accidental catalytic promiscuity) in addition to the main activity in the same active site. These low activities can be enhanced by mutagenesis and therefore provide a good starting point for protein engineering. On the other hand very similar enzymes, which belong to the same protein fold, often exhibit different enzyme activities. By comparison of the structure and the reaction mechanism of two enzymes, amino acids can be identified, the substitution of which could switch the catalytic activity of the enzyme (induced catalytic promiscuity). Some examples for increasing and introducing promiscuous catalytic activities are given below, for more extended reviews see [58–60, 57].

Based on the postulated reaction mechanism a single point-mutation was introduced into an arylmalonate decarboxylase from *Alcaligenes bronchoseptimus* to add a promiscuous racemase activity while retaining the decarboxylase activity [61]. Similarly, a detailed analysis of the reaction mechanism of the thiamine diphosphate dependent pyruvate decarboxylase identified a Glu473 residue, which was mutated and resulted in a 100-fold preference for the promiscuous carboligation reaction [62]. Hilvert and his group achieved the introduction of aldolase activity into a PLP-dependent racemase from *Geobacillus stearothermophilus* (thereby decreasing the racemase activity by >1000-fold) by mutation of just one amino acid (Y265A) in the active site, which removes a catalytic residue for the racemase activity, but creates space for the aldolase substrate [63, 64]. The aldolase activity was further extended by changing the same residue to a lysine [65]. Moreover, three additional mutations in the active site changed the preference to the (2R,3R) diastereomer relative to the (2R,3S) diastereomer. A double mutation in the active site abolished the racemase activity and increased the intrinsic forward half transaminase activity 6.6-fold in an alanine racemase from *B. stearothermophilus* [66].

**Figure 3:**
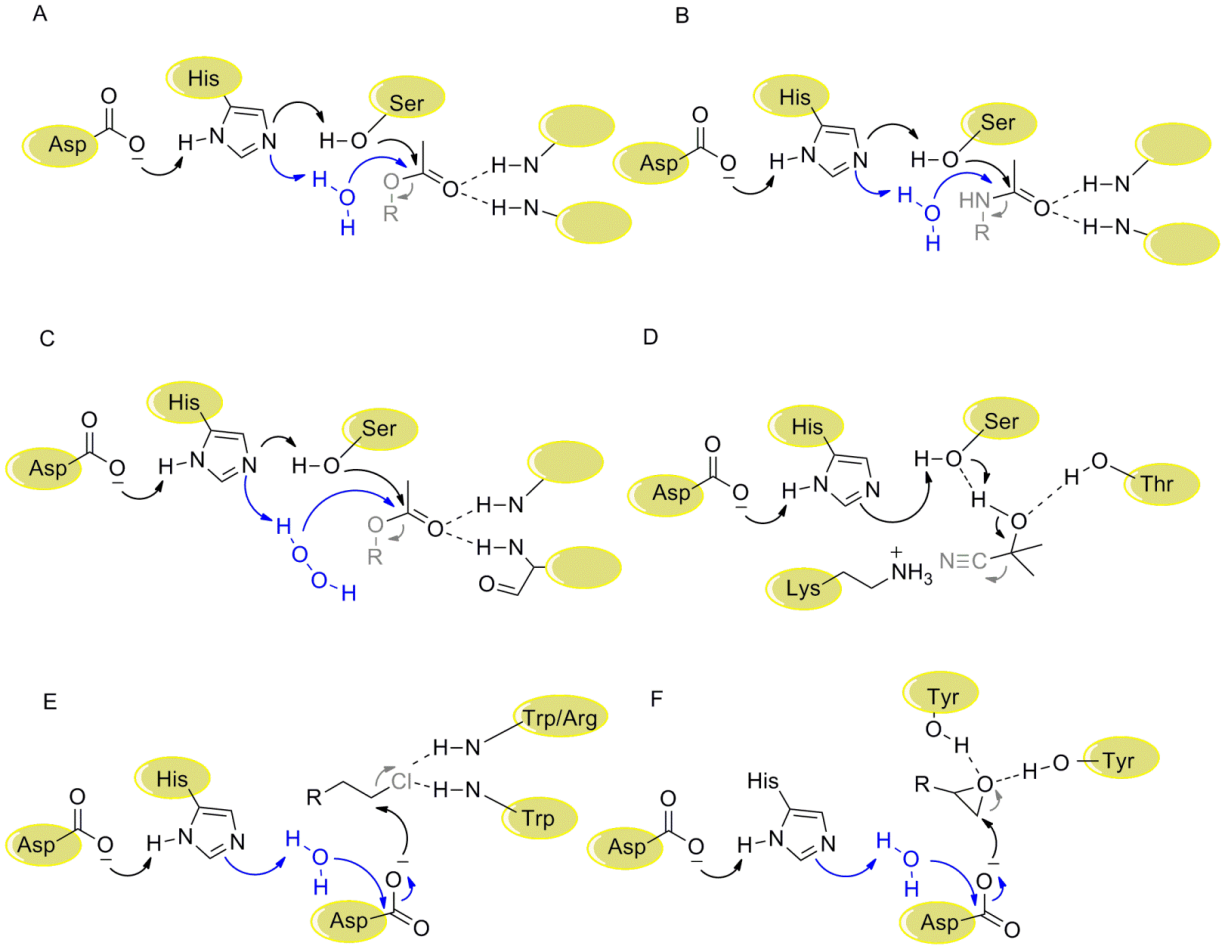
Reaction mechanism of A: esterase, B: amidase, C: perhydrolase, D: hydroxynitrile lyase, E: haloalkane dehalogenase, F: epoxide hydrolase. Esterase, amidase and perhydrolase share a common mechanism, in which a Ser, which is activated by a His-Asp dyad, serves as nucleophile. Subsequently, esterase and amidase are hydrolyzed by an activated water molecule, whereas in perhydrolases an activated H_2_O_2_ is involved. Haloalkane dehalogenase and epoxide hydrolase share a different mechanism, in which an Asp serves as nucleophile. A water molecule, which is also activated by the His-Asp dyad, hydrolyzes the ester bond between Asp and product. In contrast to these reaction mechanisms, in hydroxynitrile lyases no covalent enzyme-substrate intermediate is formed. Again the catalytic triad is involved, but the activated Ser acts as base and subtracts a proton from the hydroxyl group of the cyanohydrin. Subsequently the cyanide is released.

Enzymes with α/β-hydrolase-fold are one of the largest known protein families, including esterases, lipases, amidases, epoxide hydrolases (EH), dehalogenases and hydroxynitrile lyases (HNL) (Figure 3). Some of them already have intrinsic promiscuous enzyme activities [67].

The first example in which a promiscuous activity seemed to be increased in an esterase is the perhydrolase activity of an esterase of *P. fluorescens* [68]. The perhydrolase activity of a single-mutant (L29P) apparently increased 28-fold from 0.24 to 6.8 U/mg, while the esterase activity decreased 100-fold from 14 to 0.14 U/mg. However, different substrates (acetic acid for the perhydrolysis and p-nitrophenylacetate for the hydrolase reaction) were used for the two reactions. Recently, by the use of the same ester substrate, methyl acetate, for both reactions, it was shown that mutation L29P actually decreased the perhydrolase activity 15-fold and the hydrolysis 3-fold. Moreover, experiments revealed that the mutant did not have higher selectivity for H_2_O_2_ than for water as it was originally proposed, but that in the mutant the acyl-enzyme intermediate with acetic acid is formed faster than in the wild-type [69].

Both, esterases and some HNLs, belong to the α/β-hydrolase superfamily and have the typical conserved Asp, His and Ser catalytic triad in their catalytic site. Although the substrates and the reaction mechanisms are different, two groups managed to introduce HNL activity into esterases. Schwab and his group converted the bacterial esterase EstC from *Burkholderia gladioli* into an HNL; one mutation (S276K) was sufficient to generate HNL activity and to abolish esterase activity [70]. Kazlauskas and his group switched the plant esterase SABP2 activity by just two point mutations (G12T and M239K), resulting in an enzyme with strongly reduced esterase activity, but clearly detectable HNL activity with racemic mandelonitrile (20 mU/mg, k_cat_/K_M_ = 72 min^-1^M^-1^), but with low enantioselectivity cleaving (*R*)- and (*S*)-mandelonitrile with 12.6 and 15.5 mU/mg, respectively. In the synthesis direction, the (*S*)-product showed only 20% *ee* [71]. Comparison of the structures of epoxide hydrolases with esterases revealed differing loops at the entrance to the active site. Subsequent exchange of the loop of the esterase by an epoxide hydrolase loop finally introduced EH activity into an esterase from *P. fluorescens* (k_cat_ 0.01 s^-1^, [72]). The best variant showed high enantioselectivity (E > 100) for the (*R*)-enantiomer of *p*-nitrostyrene oxide. Some esterases already display promiscuous amidase activity. However, so far no successful shift in the reaction preference has been reported [73]. Very recently, the promiscuous enantioselective (γ)-lactamase activity of (2-azabicyclo[2.2.1]hept-5-en-3-one) in the *P. fluorescens* esterase I was increased 200-fold by the introduction of a point mutation (L29P) and the structural and mechanistic determinants for the catalytic promiscuity and enantioselectivity were identified by molecular modeling [74]. Interestingly, this is the same mutation as has been reported to play a role in perhydrolysis reaction [68] (see above). These studies together with investigations of Syren and Hult on the prerequisites for amide hydrolysis [75] might lead to conversion of an esterase into an efficient amidase.

Lipase B from *Candida antarctica* (CAL-B) was analyzed by quantum mechanical simulations for the possibility to introduce aldolase activity. Site-directed mutagenesis of Ser105 in the active site proved that very low activity for an aldol reaction was achieved [76]. Interestingly, the S105A variant is also able to catalyze Michael-type additions faster than the wild-type [77, 78]. It was suggested that the mutation of the Ser residue, which is part of the catalytic triad in the natural hydrolase activity, disrupts the hydrolase activity with no effect on the Michael-type reaction with an ester substrate [79]. Recently, docking studies of methyl 2-methyl-2-nitro-5-oxohexanoate in CAL-B S105A revealed that theoretically there is no preferred binding mode for either the (*R*)- or the (*S*)-enantiomer in the voluminous active site explaining the lack of enantioselectivity [80].

Despite the increasing number of redesigned active sites to introduce or improve promiscuous activities, most of them still lack behind natural enzyme activities. Care has also to be taken, that the apparent promiscuous reaction de facto stems from enzymatic catalysis and is not chemically catalyzed by a prosthetic group only [81] or by amino acids not related to an active site (e.g. EstB from *B. gladioli*, [82]) or from an apparent new reaction due to an overlooked change of the actual substrate e.g. hydrolysis of substrate prior to the measured reaction [83]. Thus, the choice of an appropriate method for analysis and proper controls are crucial.

### Broadening substrate range

Nowadays substrate docking is a routine method in enzyme engineering to carve the active site to improve enzyme activity, broaden the substrate scope towards industrially interesting compounds and increase or invert enantioselectivity. A few representative examples are given in this review.

In 2005, the (*R*)-selective hydroxynitrile lyase from *Prunus amygdalus* was one of the first lyases to be redesigned by modeling to give enzyme variants with improved enantioselectivity (>96%) for the synthesis of (*R*)-2-hydroxy-4-phenylbutyronitrile, which is an intermediate of angiotensin-converting enzyme inhibitors (ACEi) known as prils [84]. The crystal structure of another hydroxynitrile lyase from *Manihot esculenta* revealed that a bulky tryptophan at the entrance of the active site might be the reason for the preference of small substrates, which was confirmed by site-directed mutagenesis to a small Ala residue resulting in enhanced activity [85, 86]. Interestingly, mutation of this Trp residue to smaller residues (Ala, Met, Phe) was also identified in the highly similar HNL from *Hevea brasiliensis* by directed evolution (Schwab, unpublished results). Other examples concerning engineering of HNLs are given in other reviews (e.g. [87, 87b]). Similarly, molecular dynamics simulation and structure-based enzyme design identified key functional residues in the active site access tunnel of a dehalogenase from *Rhodococcus rhodochrous* [88]. Substitutions in these positions resulted in variants with 32-fold increase of activity towards 1,2,3-trichloropropane (TCP). This improvement was shown to originate from changes in solvent accessibility of the active site. Analysis of a transition-state model of 1-phenyl-1-hexanol in a lipase from *Burkholderia cepacia* proposed three amino acids that might improve the activity and enantioselectivity towards secondary alcohols with bulky substituents on both sites [89]. Experiments confirmed that indeed a double mutant of two of these residues showed high conversion and high enantioselectivity (E > 200, compared to E = 5 before). Analysis of the structure and molecular modeling allowed the identification of amino acids critical for switching the substrate specificity of a *Drosophila melanogaster* 2’-deoxynucleoside kinase from thymidine to 3’-deoxythymidine by side-directed mutagenesis [90]. In an approach combining *in silico* modeling, site-saturation mutagenesis and directed evolution, Savile and coworkers created an ω-(*R*)-transaminase, which is able to convert the bulky-bulky pro-sitagliptin ketone (200 g/L) to sitagliptin (an active pharmaceutical ingredient API) of >99.9% *ee* with 92% yield in 50% DMSO after eleven rounds of mutagenesis inserting a total of 27 mutations in the most active mutant [91]. The activity for aliphatic amines was improved by replacing a bulky tryptophan residue in the active site of an (*S*)-selective ω-transaminase from *Vibrio fluvialis* by a glycine to create a larger substrate binding pocket [92]. Using homology models Berglund and his group designed variants of originally (*S*)-selective ω-transaminases from *Chromobacterium violaceum* and *Arthrobacter citreus* that showed improved or reversed enantioselectivity [93, 94]. For other transaminase engineering approaches see Mathew and Yun [95]. By combination of directed and designed evolution (site-saturation mutagenesis of three amino acids in the active site, that have been identified in the structure) of the esterase EstB from *B. gladioli* the enantioselectivity was inverted [96]. Simultaneous site-saturation mutagenesis of two residues in the active site of esterase B2 from *B. subtilis* resulted in inverted enantioselectivity from E_R_ > 100 to E_S_ = 65 [97].

Many oxidoreductase enzymes are NAD(P)H dependent. In pathway engineering of microorganisms used for the production of industrially relevant products the balance of cofactor consumption and regeneration is essential to obtain a high yield. Structure-guided site-specific mutagenesis to change the cofactor preference has been reported several times e.g. in xylose reductases [98, 99], phosphite dehydrogenase [100], alanine dehydrogenase [101], (BVMO) phenylacetone monooxygenase [102] and many more. As nicotinamide cofactors are expensive, they are frequently recycled in biotechnological processes, e.g. by the use of NAD(P)H oxidases. Bommarius and his group engineered the substrate binding pocket of an NADH oxidase from *Lactobacillus plantarum* by site-specific mutagenesis to accept also NADPH [103].

### *De novo* enzyme design

Novel enzymes can be either designed by recreating known enzymatic functions in proteins with a different fold or by introduction of activities that have not been observed in natural enzymes before into a chosen protein scaffold. The biggest challenge, however, is the design of *de novo* enzymes that are not based on natural sequences, thus designing the complete protein from scratch [105–108]. This is beyond the scope of this review and will not be addressed in detail. Most *de novo* enzyme design approaches depend on computational methods, which have been developed and improved in the last two decades [108, 109, 6, 106, 111–113].

**Figure 4:**
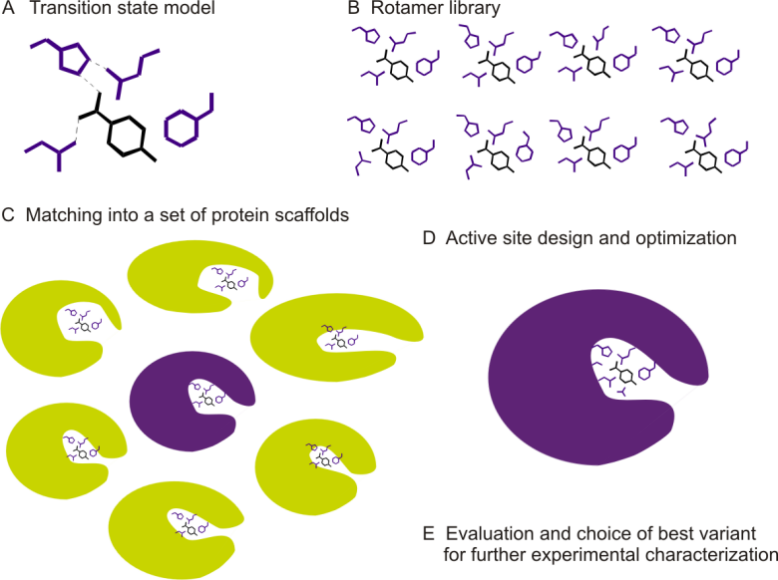
*De novo* design of active sites in existing protein scaffolds.

For the approaches described here, a detailed knowledge of the reaction mechanism and transition state is crucial to be able to predict which amino acids at which positions and distance are necessary to form an active site and catalyze the desired chemical reaction. In an approach by the Baker lab, in the first step idealized models of an enzymatic transition state are created by the use of quantum mechanical calculations (theozymes), which are in the next step fitted into protein scaffolds, which were identified from a library of folds and can accommodate the amino acids of the active site without clashes (RosettaMatch) [113, 114]. However, usually no perfect match is found and thus subtle deviations from the theozyme geometry have to be accepted (Figure 4). Another possibility offers SABER (Selection of Active/Binding sites for Enzyme Redesign), a computational method developed by the Houk lab, which searches for structures in which the necessary amino acids are already in place and only the substrate needs to be fitted in its transition state geometry [115]. Finally, in both approaches, the surrounding side chains are optimized for favorable interactions with the substrate/transition state model and stabilization of the protein fold (RosettaDesign) [113, 114].

The most prominent examples for designed novel enzymes with non-natural enzyme activities are a Kemp eliminase, a Diels-alderase and a retroaldolase. Although currently enzymes, which are designed *de novo* by computational methods don't reach the performance of natural enzymes or variants evolved in the laboratory, in some cases, they can be improved by standard protein engineering methods [5, 116]. Mechanistic studies and structural analysis (also of inactive designs) will give indications about the quality of the design, identify problems, and help to improve the model and the computational parameters [117–124].

The computationally designed retroaldolase utilizes a catalytic lysine residue that forms a Schiff base with a keto group on the substrate, and then serves as an electron sink during C-C bond cleavage. To date 65 active designs were constructed in 14 different protein scaffolds [124, 125], however with very low starting activities (k_cat_/K_M_ values of less than 1 M^-1^s^-1^), which were further improved by systematically mutating amino acids at or near the active site (improve of k_cat_/K_M_ values up to 88-fold) and directed evolution (in one protein the activity improved 700-fold over ten evolutionary cycles, a combination of a 100-fold increase in k_cat_/K_M_ and an increased yield of soluble protein) [124, 121]. However, the best improved and evolved retroaldolase design reaches a k_cat_/K_M_ value of 55, which is still below catalytic antibodies with k_cat_/K_M_ value of 490 under the same reaction conditions [124].

Other examples in which the initial design was further improved by random mutagenesis are the Kemp eliminase KE07 (improvement of k_cat_/K_M_ values from 12 to 2,600 M^-1^s^-1^) [126] and the variant KE70, which was improved by a combination of rational and random mutagenesis to k_cat_/K_M_ values up to 5x10^4^ M^-1^s^-1^ [127]. The initial design combined a catalytic base, either Asp or Glu (KE07), or a His-Asp/Glu dyad (KE70), a hydrogen bond donor (Ser or Lys) to stabilize the accumulating negative charge that develops on the phenolic oxygen, and an aromatic residue for substrate binding and delocalization of the negative charge of the transition state. Of the 59 final designs, only eight showed activity [128]. Interestingly, the initially catalytically most active design KE59 was one of the most instable variants. After introduction of fold-stabilizing consensus mutations and 16 rounds of directed evolution the k_cat_/K_M_ value increased > 2000-fold (from 163 to up to 0.6x10^6^ M^-1^s^-1^) due to a large improvement of k_cat_ [129]. Moreover, the optimized variant has a broader substrate acceptance. Structural analysis and MD simulations of improved variants of KE07, KE70 and KE59 led to explanations for the higher activity including a tighter substrate binding and the stabilization of the active-site dyad (KE70) in a conformation optimal for catalysis and a general more soluble and stable protein. In a different strategy of the Houk and Mayo labs - instead of characterizing many different designed proteins - the active site of a single template protein was redesigned to a Kemp eliminase using an approach described before [130]. After a detailed analysis of the structure and dynamics of an inactive first design and identification of possible causes for the inactivity, the focus of the design was moved from the native active site of the protein to a small pocket deeper in the protein [131]. This second design contained twelve mutations and resulted in a Kemp eliminase with a k_cat_/K_M_ value of 123 M^-1^s^-1^, which was further improved 3-fold by single point mutations. Interestingly, two other groups managed to introduce Kemp eliminase activity into a hydrophobic pocket of calmodulin and a buried cavity in T4 lysozyme (differing from the native active site) by the introduction of just one charged amino acid, Glu and His respectively; however exhibing lower activity (k_cat_/K_M_ values of 5.8 and 1.8 M^-1^s^-1^, respectively) than the above mentioned designs [132, 133]. The k_cat_/K_M_ values of catalytic antibodies for Kemp eliminations were reported to be 5,500 M^-1^s^-1^ using carboxylate as general base [134] and what is even more intriguing serum albumins e.g. BSA can catalyze the same reaction with k_cat_/K_M_ values of 2,600 M^-1^s^-1^ by the action of a lysine side chain in a hydrophobic environment [135].

An even bigger challenge was the computational design of a Diels-Alderase as it catalyzes the intermolecular C-C bond formation of two substrates [136]. Of the initially 10^19^ variants calculated by QM simulations, 10^6^ could be fitted into protein scaffolds and after further optimization finally 84 designs were chosen for biochemical characterization, of which 50 were expressed as soluble proteins but only two showed the desired activity. Although the turnover rate is very low (2 s^-1^), one variant is highly substrate- and stereospecific forming >97% of the stereoisomer (out of 8 possible) of the substrate it was designed for. An interesting approach was chosen for further improvement of this designed Diels-Alderase variant by challenging the players of the online computer game Foldit, which was created by the Baker Lab to help to predict protein structures [137], to remodel the active-site loops to enable additional interaction with the substrate [138].

The most recent *de novo* design is the introduction of a catalytic Cys-His dyad and oxyanion holes for ester hydrolysis into different protein folds [139]. The best design so far, which was further improved by site-specific mutagenesis reached a k_cat_/K_M_ value of 405 M^-1^s^-1^, which is still lower than catalytic antibodies. Again structural analysis revealed bottlenecks, which will help to improve subsequent designs.

## Artificial metalloenzymes

Artificial metalloenzymes are thought as a bridge between biocatalysis and transition-metal complexes by incorporating the catalytically active transition metal complex in the protein scaffold enabling a high activity and selectivity [105, 140]. There are several approaches how this can be achieved, which can be divided in two main categories: non-covalent anchoring, where either the affinity of a protein for a transition metal is used (dative anchoring) or high-affinity protein substrate interactions are employed (supramolecular anchoring), and covalent modification (for a review see [141]). The range of metal ions that can be incorporated in the active site increases the range of chemical transformations catalyzed by the enzyme. Some enzymes already display promiscuous activity due to different metal ions present in the active site (e.g. [142, 143]). In some cases the intrinsic metal ions of natural metalloproteins were successfully exchanged to different metal ions, thereby altering the reaction catalyzed by the enzyme. Carbonic anhydrase II is a zinc metalloenzyme with Zn^2+^ interacting with three His residues. Its physiological role is the catalysis of the reversible hydration of carbon dioxide with a k_cat_/K_M_ value close to the diffusion limit. However, carbonic anhydrase II shows also promiscuous esterase activity [144]. The replacement of the Zn^2+^ in human carbonic anhydrase by Mn^2+^ resulted in a peroxidase, which catalyzes the efficient oxidation of o-dianisidine (k_cat_/K_M_ = 1.4x10^6^) in the presence of bicarbonate and hydrogen peroxide (H_2_O_2_), and also epoxidation of olefins, however with only low to moderate enantioselectivity [145, 146]. The same group also showed that rhodium-substituted carbonic anhydrase induced stereoselective hydrogenation of stilbene favoring *cis*- over *trans*-stilbene by about 20:1 [147]. It also catalyzes the hydroformylation of olefins with a regioselectivity of 8.4 for linear over branched aldehyde product, which is in opposite to uncomplexed Rh, which results in mainly branched product [148]. Exchange of the metal ion in the Zn^2+^-dependent β-lactamase from *Stenotrophomonas maltophilia* to Cu^2+^ resulted in 10-fold lower lactamase activity and a promiscuous new oxidase activity towards catechol. This oxidase activity was further improved by site-specific mutagenesis to stabilize the coordination of the Cu^2+^ ion in the second metal binding site [149].

Another option is the creation of a novel metal binding site in a host protein by introduction of coordinating amino acids at geometrically appropriate positions of the protein. Reetz and his group introduced two His residues in the thermostable protein tHisF, the synthase subunit of an imidazole glycerol phosphate synthase, from *Thermotoga maritima* to create a 2-His-1-carboxylate motif for the binding of Cu^2+^ [150]. They showed that the enantioselectivity of a model Diels-Alder cycloaddition was enhanced with the protein-Cu^2+^ catalyst compared to Cu^2+^ alone. A similar approach was followed by Lu and coworkers, who introduced three histidines and one glutamate or two histidines and one glutamate to create a new non-heme iron center in myoglobin near the unchanged heme cofactor to design a nitric oxide reductase (NOR) [152–154], which should help to understand the reaction mechanism of naturally occurring NORs. Both enzymes converted NO to N_2_O. Myoglobin was found to display weak nitrite reductase (NiR) activity. The X-ray structure of NiR identified the location of two His and one Tyr residues, which are essential for NiR activity. As for NOR, suitable target residues for mutagenesis were identified in myoglobin by molecular modeling in combination with molecular dynamics simulations giving an insight into the nitrite binding by calculation of theoretical K_M_-values [154]. However, so far experimental data are missing.

In yet a different strategy mononuclear zinc metalloenzymes were computationally redesigned to catalyze the hydrolysis of organophosphates by changing active site amino acids while maintaining the coordination geometry around the metal ion [155]. Of twelve designs a redesigned adenosine deaminase hydrolyzed the achiral organophosphate diethyl 7-hydroxycoumarinyl phosphate (DECP) and was further improved by directed evolution (k_cat_/K_M_ values up to 10^4^ M^-1^s^-1^). It also hydrolyzed the coumarinyl analog of the nerve agent cyclosarin with a preference for the R_P_ isomer.

## Conclusion

This review describes recent advances in enzyme engineering focusing on rational approaches for the creation of biocatalysts with improved activity, enantioselectivity, substrate range, stability and also new catalytic functions. It is highlighted that in many cases protein structures helped to understand the basis of the enzyme's reaction mechanism and allowed the successful fine-tuning of the active site by rational design by only a few specific mutations thereby providing a strong alternative to extensive screening of large libraries with directed evolution approaches.

However, despite the availability of a fast-growing number of protein structures and highly sophisticated computational algorithms pure rational design is still limited by (i) our incomplete understanding of the enzyme functions and protein folding, (ii) the flexibility of the proteins and conformational changes upon e.g. substrate binding, and thus (iii) our still limited understanding of protein dynamics [122], (iv) the sensitivity of the enzymatic reaction to small changes in distances and geometry of the substrate in relation to the active site amino acids and to the influence of active site water molecules [139, 123], (v) the adverse effect of some mutations on the expression and stability of the protein (e.g. [156, 157]), (vi) the positive effect of apparently unrelated distant mutations as well as correlated mutations, which are at the moment difficult to predict [158, 40, 17, 24] and (vii) the enormous computational capacity needed for processing large data volumes.

Thus, pure computational design of *de novo* enzymes is still in its infancies and the tailored biocatalysts show just small, but promising activities. However, the probability of the success of solely random strategies is also low and requires high screening effort. This review provides several examples, in which the combination of rational design with subsequent directed evolution led to the most promising *de novo* enzymes.

In summary, there exists no general strategy how to proceed in the engineering of a certain enzyme. It strongly depends on the reaction and enzyme of interest, the biochemical and structural data available, the structural bioinformatics expertise and computational equipment, and the library screening capabilities to mention just a few. Currently, computational enzyme design has fundamentally changed the way biocatalysts can be altered, but it cannot yet replace directed evolution as a method of choice for protein engineering. On the contrary, the two approaches are complementary and should be combined for reaching the most promising results either in semi-rational approaches or using them subsequently.
